# Symmetry-adapted pair distribution function analysis (SAPA): a novel approach to evaluating lattice dynamics and local distortions from total scattering data

**DOI:** 10.1107/S1600576721008499

**Published:** 2021-09-13

**Authors:** Tobias A. Bird, Anna Herlihy, Mark S. Senn

**Affiliations:** a Department of Chemistry, University of Warwick, Gibbet Hill, Coventry CV4 7AL, United Kingdom; b ISIS, Rutherford Appleton Laboratory, Chilton, Didcot, Oxfordshire OX11 0QX, United Kingdom

**Keywords:** PDF, pair distribution functions, symmetry-adapted approaches, soft modes

## Abstract

A novel symmetry-adapted pair distribution function analysis (SAPA) method for extracting information on local distortions from pair distribution function data is introduced. The implementation of SAPA is demonstrated in the *TOPAS-Academic* software using the freely available online software *ISODISTORT*, and scripts for converting the output from *ISODISTORT* to a SAPA input file for *TOPAS* are provided. Finally, two examples are provided to show how SAPA can evaluate the nature of both dynamic distortions in ScF_3_ and the distortions which act as an order parameter for the phase transitions in BaTiO_3_.

## Abstract 2

A novel symmetry-adapted approach to analysing X-ray and neutron pair distribution function data is presented. Local deviations from the average structure are determined using representation analysis.

## 1. Introduction   

The technique of total scattering, by which one obtains a pair distribution function (PDF), is an increasingly powerful tool used to analyse the local structure of a variety of materials (Keen, 2020 ▸). The first quantitative measurements using this technique were made in the 1930s, when experiments were performed on liquid mercury (Debye & Menke, 1930 ▸) and sodium (Tarasov & Warren, 1936 ▸). Since this early work, the technique has seen a wide range of uses, such as comparing crystalline and amorphous structures of the same materials (Biscoe & Warren, 1938 ▸; Hultgren *et al.*, 1935 ▸; Warren *et al.*, 1936 ▸; Peterson *et al.*, 2013 ▸), modelling crystalline disorder (Keen *et al.*, 2005 ▸; Senn *et al.*, 2016 ▸), and studying the dynamics of more ordered materials (Bird *et al.*, 2020 ▸; Conterio *et al.*, 2008 ▸; Goodwin *et al.*, 2009 ▸). The focus of this work is to present a novel approach for the latter. Whilst there are more established methods of investigating phonons in crystalline materials, chiefly inelastic neutron scattering, these methods require single crystals and are often quite time consuming. In comparison, total scattering experiments are relatively easy to perform, only require a powder sample and are more time efficient. There are now even laboratory-based instruments that can collect X-ray total scattering data, making it a more readily available technique (Confalonieri *et al.*, 2015 ▸; Thomae *et al.*, 2019 ▸; Irving *et al.*, 2021 ▸).

The PDF of a material is obtained via a Fourier transform of the observed scattering function *S*(*Q*) (Keen, 2020 ▸). The scattering function contains structural and lattice dynamics information, and therefore this information should also be present in the PDF. Indeed, it has been shown that experimental PDF peak widths correlate well with mean-square displacements obtained from lattice vibration models (Jeong *et al.*, 1999 ▸, 2003 ▸).

Various methods have been used to try and retrieve the dynamic information from the PDF. The first method used standard phonon models of the studied materials. By comparing their associated PDFs with those observed experimentally, reasonably accurate dispersion curves could be reproduced for some fairly simple systems such as face-centred cubic rhodium (Dimitrov *et al.*, 1999 ▸), but the process became increasingly inaccurate when more parameters were required in the phonon model (Reichardt & Pintschovius, 2001 ▸; Graf *et al.*, 2003 ▸). The second method used the reverse Monte Carlo (RMC) method to produce a large number of atomistic configurations, which can be thought of as snapshots of the material at different times since they are all consistent with the input PDF (Goodwin *et al.*, 2004 ▸, 2005 ▸, 2009 ▸; Conterio *et al.*, 2008 ▸). These configurations can then be used to construct a phonon dispersion curve *via* methods developed for molecular dynamics simulations. The same authors have used a similar technique to construct spin-wave dispersion curves (Goodwin *et al.*, 2007 ▸). This method has been shown to produce reliable information for low-frequency modes but fails to reproduce higher-frequency features, such as longitudinal-optical/transverse-optical mode splitting. This is to be expected, since the Bose factor in the phonon cross section, which approaches 1/ω^2^ at higher temperatures, means that the PDF is much more sensitive to low-frequency information. A later paper used a similar method to look at disorder in BaTiO_3_ and Bi_2_Ti_2_O_7_, but employed representational analysis rather than molecular dynamics methods to quantify the dynamics (Neilson & McQueen, 2015 ▸). Whilst these methods have their uses, they both have some downsides. They are both computationally intensive and require a lot of modelling to produce any results. In addition, the RMC method requires a more intensive setup process than the method presented here. The first method also requires preselection of a phonon model and hence an assumption of the nature of the local distortions, introducing bias into the analysis. As a result of this, there have been relatively few papers using either method.

The method presented here, symmetry-adapted PDF analysis (SAPA), involves expanding the possible degrees of freedom of the crystallographic unit cell up to a given supercell size in terms of symmetry-adapted displacements of the zone centre and zone boundary irreducible representations (irreps) of the structure being studied, or a higher-symmetry parent structure. The collection of symmetry-breaking displacements transforming as the same irrep may be further decomposed into symmetry-adapted distortion modes by choosing a sensible basis that reflects the chemistry and crystallographic axes of the structure. The distortion modes have a 1:1 correspondence with phonon eigenvectors in the limit that only one set of atomic displacements transforms as the corresponding irrep. It is hence justifiable (in the harmonic approximation) to test distortions belonging to a given irrep against the data in turn. In cases where distortions from different Wyckoff sites transform as the same irrep, the character of the low-lying excitations can still be ascertained through refining the relative amplitudes of the individual distortion modes simultaneously. This method does not aim to produce a dispersion curve from diffraction data; the goal is to determine which of the symmetry-adapted distortion modes are most responsible for local deviations from a parent or average structure. The method has been used successfully to study order–disorder-type phase transitions in BaTiO_3_ (Senn *et al.*, 2016 ▸) and the dynamic distortions responsible for the large magnitude of negative thermal expansion in ScF_3_ and CaZrF_6_ (Bird *et al.*, 2020 ▸). The process itself is similar to one presented by Kerman *et al.* (2012 ▸) to determine the average structure of a distorted material, although the primary aim of the SAPA method is to determine how the local structure deviates from the average.

## 2. Method   

The primary tool presented in this paper is a script to convert mode parameterizations generated by the *ISODISTORT* software (Campbell *et al.*, 2006 ▸) into pair distribution function refinement input files for the *TOPAS-Academic* software (Coelho *et al.*, 2015 ▸). This script groups the symmetry modes in the input file with respect to the irreducible representation they transform as, and allows these groups of modes to be turned on in refinements from the command line.

### 2.1. Software   

This method uses the *TOPAS-Academic* software v6 (Coelho, 2018 ▸) with an additional set of macros and functions for use with pair distribution function refinements. These macros and functions are obtained by downloading the pdf.inc file from the GitHub repository of Chater (2017 ▸). A strength of the *TOPAS* software is that it is written in its own scripting language. This has allowed users from the scientific community to implement new methods, including symmetry-mode refinements (Lewis *et al.*, 2016 ▸). Additionally, the method it uses to refine PDF data is significantly faster than alternative programs (Coelho *et al.*, 2015 ▸), including the *PDFfit2* program (Farrow *et al.*, 2007 ▸), in which this symmetry-adapted PDF analysis method was initially implemented (Senn *et al.*, 2016 ▸). The online tool *ISODISTORT* (version 6.9.0, June 2021) was used to generate the mode parameterizations.

### 2.2. Generating mode parameterizations   

**Figure 1:**
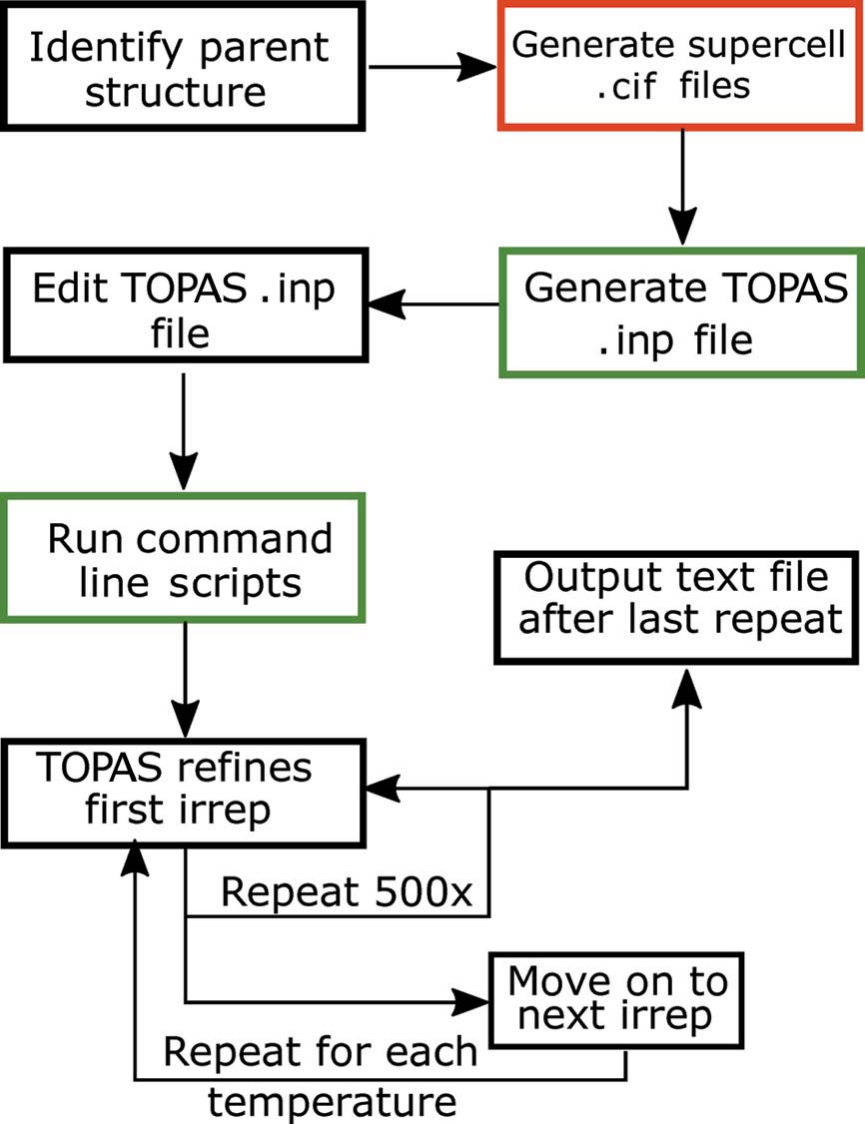
A diagram showing the processes to go through to use the expanded small box method. Steps in a red box use the online tool *ISODISTORT* and steps with a green box use the Python programming language.

An overview of the steps required to perform a symmetry-adapted PDF analysis is shown in Fig. 1 ▸. The first step in the process of performing this symmetry mode analysis is to identify the parent structure to be used. When analysing the dynamic distortions of a material that stays in the same phase over the temperature or pressure range of interest, that phase can be chosen as the parent structure. For example, when performing this analysis on the negative thermal expansion material ScF_3_, which retains its cubic 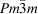 structure down to very low temperatures, the 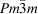 phase was chosen to perform the analysis. If however the material undergoes phase transitions, or stays in one phase but is a distorted version of a higher-symmetry parent structure, the undistorted parent structure would be a sensible choice. For example, to study a distorted perovskite, or a perovskite which undergoes a phase transition in the temperature range of interest, the aristotype perovskite structure could be used.

The next step is to decide the supercell size to expand up to. The modes that will be included and excluded when choosing the supercell must be considered; for example, a 3 × 3 × 3 supercell would not include distortions with propagation vectors of [1/2 0 0], [1/2 1/2 0] or [1/2 1/2 1/2]. Another element of the analysis to keep in mind is that expanding the structure further increases the number of modes and therefore increases the time it takes to get results. For example, the 2 × 2 × 2 supercell of ScF_3_ contains 32 atoms and therefore has 96 distortion modes. Increasing the supercell to 3 × 3 × 3 increases the number of atoms to 108 and the number of modes to 324. Furthermore, distortion modes corresponding to very low symmetry points of the Brillouin zone will not benefit from any symmetry constraints on their characters. In addition, their long-wavelength nature will mean that low-*r* regions of the PDF will not contain sufficient information to constrain them.

Once the previous two steps have been completed, the mode listing can be generated using the *ISODISTORT* program. Firstly, a .cif file of the chosen parent structure must be imported, taking careful note of the setting and positions of the atoms used in this structure since they can have an effect on the assignment of irrep labels in the analysis. The *ISODISTORT* option ‘Method 3: Search over arbitrary k points for specified space group and lattice’ is then used. In order to include all possible distortion modes, the user should select *P*1 space-group symmetry and then input the supercell size by changing the diagonal elements in the representative basis: *e.g* if choosing a 2 × 2 × 2 supercell, *a*′ = 2*a* + 0*b* + 0*c*, *b*′ = 0*a* + 2*b* + 0*c* and *c*′ = 0*a* + 0*b* + 2*c*. Clicking ‘OK’ here opens a new window prompting the user to finish selecting the distortion mode. There should only be one option here, so the user should just click ‘OK’. The next page will have a list of all the distortion modes grouped by irrep and a few options at the top. On choosing the ‘CIF file’ option and again clicking ‘OK’, the user will be prompted to save a .cif file – it is recommended not to include spaces or special characters in the file name, as this could cause later steps to not work.

**Figure 2:**
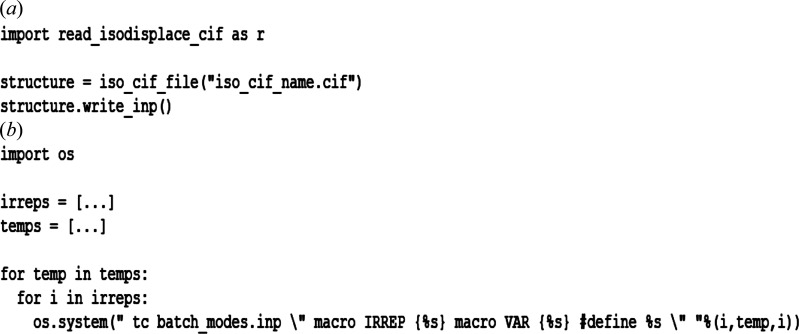
A snippet of Python code showing (*a*) how to use the write_inp method of the read_isodisplace_cif script to generate a *TOPAS* input file and (*b*) how to use the Python os library to run the generated file on the command line. Here, irreps and temps are user-defined lists.

The majority of the *TOPAS* input file can be written using information from the CIF produced with *ISODISTORT* in the previous step. To make this easier, we have written a script (available on an online repository; Bird & Senn, 2021 ▸) in the Python programming language which can read a CIF and convert it to a Python class. The data names defined in the CIF are accessible as class variables in Python. A method of this class, write_inp, uses this to output a *TOPAS* .inp file. A snippet of code demonstrating how to use this is shown in Fig. 2 ▸(*a*). In this section, we will go through the contents of the .inp file and detail any information that the user has to input. Another method of this class, irrep_list, can be used to produce a list of irreps in the .cif file, which is useful when running the .inp file. Note that this script will not work without the string #End at the end of the CIF. Files generated using *ISODISTORT* should have this already included, but those generated from other sources may not. In addition, the user should ensure that there is no white space at the start of each line.

**Figure 3:**
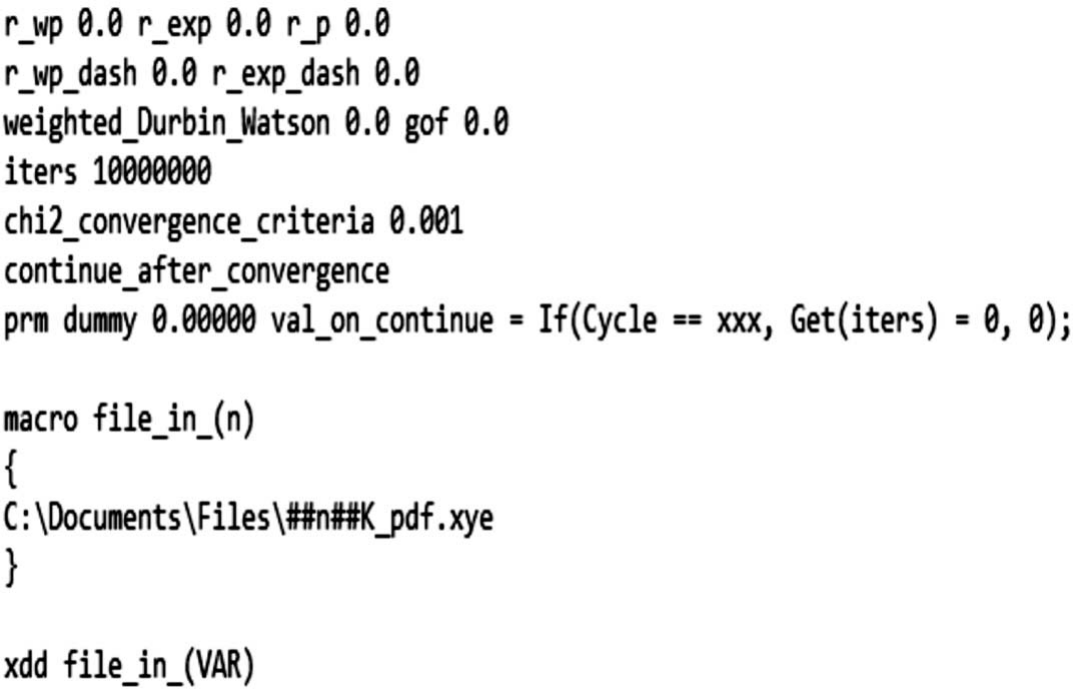
The start of a *TOPAS* input file for analysing a PDF via symmetry-mode analysis. xxx should be replaced with the desired number of repeat refinements for each irrep. In the present work, xxx = 500 has been employed to ensure that the global minimum has been reached. In addition, the directory here is taken to be the directory in which the data are stored. The string ##n## is replaced by the command line macro according to the naming convention of the variable-temperature data sets.

The first thing the user must decide is the number of cycles that *TOPAS* will perform for each irrep. At the start of each cycle, the mode amplitudes for the defined irrep are randomized within a set range *via* the continue_after_convergence and val_on_continue commands. For irreps that have a higher dimensionality, more cycles are needed to ensure that the global minimum is found for that irrep, but this has the trade-off of increasing the time needed for a full set of refinements. A good starting point is to choose 500 cycles. We found that, on a standard single-core laptop computer, 500 repeat refinements of a single irrep tended to take about 20 min. The command to specify a fixed number of cycles is shown in the seventh line of Fig. 3 ▸. The second part of Fig. 3 ▸ is the file input macro. This can be used when the data files are in a consistent format. The macro itself should reflect the user’s directory structure. The macro is called in the xdd line of the input file, and the VAR keyword is updated with a user-defined variable in the command line, which is explained in more detail in the next section. To make things easier, it is recommended to rename the data files to reflect the variable(s) that are changing with each PDF. For example, the files for the ScF_3_ example below reflect the temperature that the data were collected at.

Following this, the user must input some functions to take care of any instrumental and processing factors in the PDF. All of these functions, and others necessary for this method, can be found at the cited GitHub repository (Chater, 2017 ▸). The first instrumental function is to model the damping effect of the reciprocal-space peak width, d*Q*. If the Bragg peaks can be modelled well with a Gaussian peak shape, then the function dQ_damping should be used. If the Bragg peaks have a significant Lorentzian component, then this can be modelled using the dQ_lor_damping function. If the Bragg peak width increases as a function of *Q*, which results in *r*-dependent broadening in the PDF, the convolute_alpha function can be used. This does significantly slow down refinements however, and including it typically does not affect the qualitative output for this method. All three of these parameters (d*Q*, the Lorentzian contribution to the Bragg peak shape and the linear peak width scaling) can be refined from the Bragg data of a standard using the peak shape function pkshape_dQ_alpha. The termination ripples in the PDF originating from a finite *Q*_max_ can be accounted for using the convolute_Qmax_Sinc function. This function fails at low radii, so it is recommended to limit the refinement range to above 1 Å. If a Lorch or Soper–Lorch function has been used to mitigate against termination ripples prior to the Fourier transform of *S*(*Q*), then the convolute_Lorch or convolute_SoperLorch function should be used instead. The usage of these functions is detailed fully in the pdf.inc file. All of the values set at this stage of the input file should be fixed when running the file.

The major choice the user has to make when creating the input file is the PDF peak shape function. In Bragg scattering, the thermal motion is assumed to be completely uncorrelated, so a Gaussian function that ignores any correlated displacements is a reasonable approximation, and a constant value *B*_iso_ can be used to account for the thermal motion of an atom. In PDFs, this is not a good approximation, since atoms which are closer together will tend to have highly correlated motion, resulting in a narrower peak width at low radii. This kind of correlated motion is precisely what we aim to extract with this symmetry-motivated approach for analysing PDFs. To account for this, the single value *B*_iso_ (beq in *TOPAS*) is replaced with a radius-dependent function. There are a variety of options to choose from for this function, all of which are defined in the pdf.inc file. The simplest is the beq_rcut function, which is a step function between two constant values, increasing from the smaller to the larger at some defined cut-off radius. Another simple function, beq_spherical, uses the PDF of a sphere to scale between a value at low radius and another at a higher radius. The beq_rcut_rlo_spherical, beq_rlo_spherical and beq_rcut_spherical functions use a combination of cut-offs and spherical scaling. The function that is included by default by the write_inp method is beq_r_r2, which is a quadratic function of the radius, *r*. We find that the coefficient of *r* ^2^ refines to negligible values, so typically fix it at zero. It is advisable to choose a simple peak width function – the symmetry-adapted displacements being refined will account for some of the peak width, and introducing more parameters can lead to undesirable correlations. The *PDFfit* peak shape function is also implemented. However, we have found that refinements are often unstable when using it and, in particular, refinements with a constrained order parameter direction often fail to find the global minimum.

The last thing in the input file is the file output macro. Similarly to the file input macro, the VAR and IRREP keywords are replaced in the command line. This function, used in conjunction with the out_prm_vals_on_convergence command, produces an output file for each irrep and each temperature or pressure which has a record of the final values for each cycle for all refined variables in the input file.

### 2.3. Running the input file   

The input file is intended for use with the *TOPAS* command line executable, which requires that the working directory is the directory where *TOPAS* is installed. The input file uses #ifdef and #ifndef directives in conjunction with the #define directive, the last of which can be passed on the command line, to refine each irrep in sequence. While all the symmetry modes belonging to the irreps are defined in the input file, the user can choose which modes, grouped by irrep, to activate. The *TOPAS* command line executable also has the ability to replace user-defined keywords in the input file with values passed on the command line. For example, writing macro VAR {X} on the command line will replace the keyword VAR with the value *X* wherever it is found in the input file. To use the input file, the user must define a list of temperatures or pressures to sequentially replace the VAR keyword with and a list of irreps to cycle through. Example scripts to execute the generated input file are included in the online repository (Bird & Senn, 2021 ▸) and a demonstration of how to run the input file is given in Fig. 2 ▸(*b*).

A successful execution of the input file will produce a series of output files. A separate output file is produced for each irrep and each temperature, containing the *R*_wp_ and the value of every refined parameter for each cycle in the order they were performed. These files are delimited by white space and can be analysed by standard data analysis software packages such as *R* (https://www.r-project.org/) or the *pandas* library for Python (https://pandas.pydata.org/).

## 3. Examples   

The files for both examples, including example input files and Python scripts to execute the analysis, are included in the online repository (Bird & Senn, 2021 ▸).

### 3.1. Scandium trifluoride   

Scandium trifluoride (ScF_3_) is a material that exhibits isotropic negative thermal expansion (NTE) over a wide temperature range and is typically used to demonstrate the rigid unit mode (RUM) model of NTE. It is formed of corner-sharing ScF_6_ octahedra and remains in a 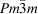 cubic structure as the temperature is lowered to 0 K. Owing to its high-symmetry structure, lack of phase transitions and interesting dynamics, it makes a good test case on which to apply this symmetry-adapted PDF analysis. Since ScF_3_ remains in the same space group, the choice of the 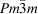 phase as the parent structure for the analysis is trivial – the only choice to make is the structure setting, which determines the irrep labels. For this work, the setting with Sc at 1*b* (1/2, 1/2, 1/2) and F at 3*c* (0, 1/2, 1/2) was used. A 2 × 2 × 2 supercell was chosen for the unit-cell expansion, since this allows phonon modes with propagation vectors **k** = [0 0 0], [1/2 0 0], [1/2 1/2 0] and [1/2 1/2 1/2] to be modelled and distortions with these **k** vectors are very common in perovskite and perovskite-adjacent compounds like ScF_3_. This study uses X-ray PDF data generated from total scattering data collected at the P02.1 beamline at PETRA III, DESY, Germany. *Q*_max_ = 21 Å^−1^ was used with d*Q* = 0.08 Å^−1^. Complete experimental details can be found in our previously published work (Bird *et al.*, 2020 ▸). An example input file is included in the online repository (Bird & Senn, 2021 ▸). The input file contains definitions for modes belonging to all the high-symmetry irreps of the 2 × 2 × 2 supercell. However, use of the #ifdef and #ifndef directives in conjunction with the #define directive allows the modes belonging to a single irrep to be refined with all the others ‘turned off’.

**Figure 4:**
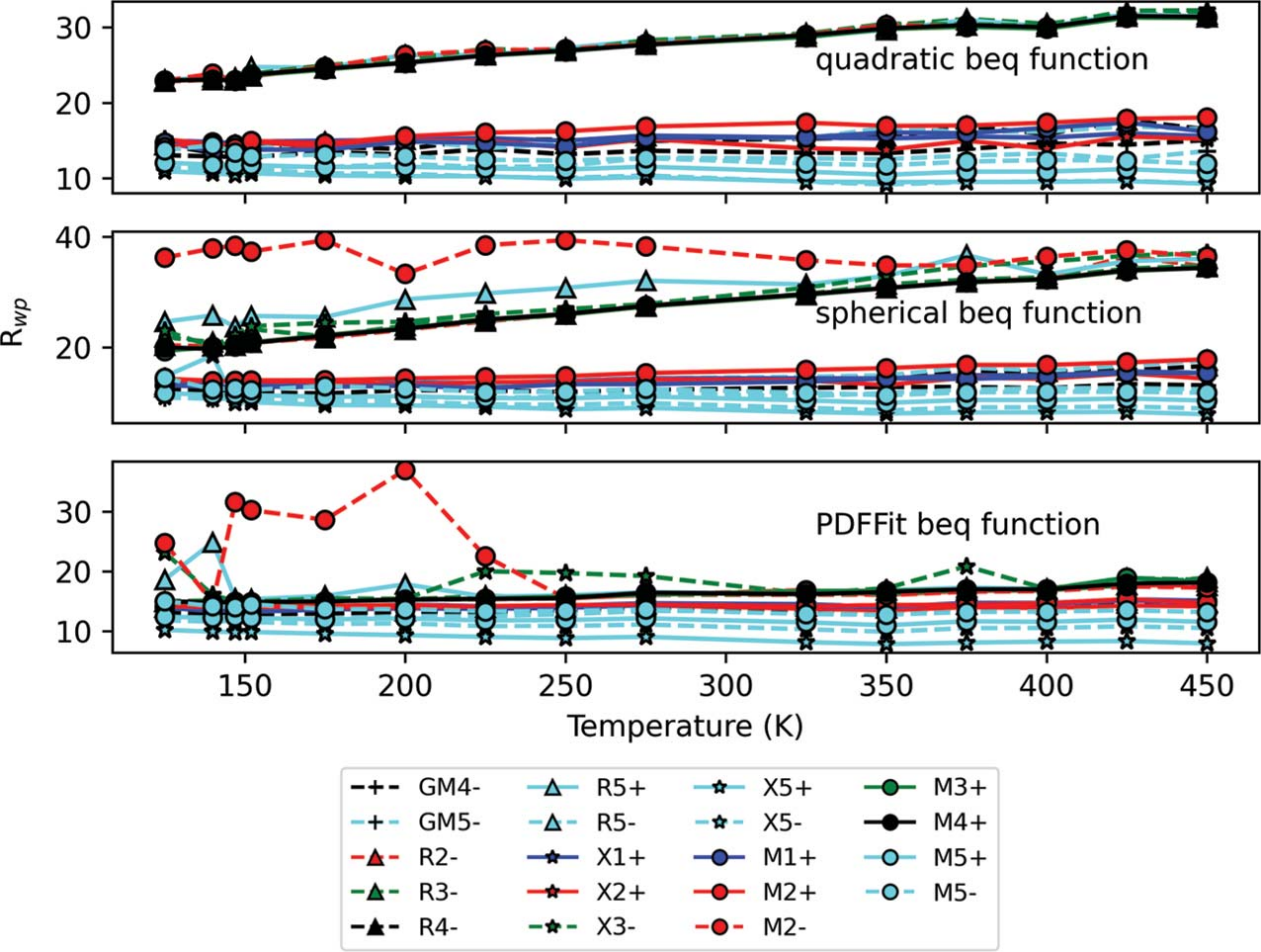
A plot of the best *R*_wp_ of all irreps against temperature for three different PDF peak shape functions used during refinement against ScF_3_ X-ray PDF data. The data points are made to differentiate between irrep labels: the marker designates the *k* point (a circle for M, a triangle for R, a star for X and a plus for Γ); the colour designates the number in the subscript (blue for 1, red for 2, green for 3, black for 4 and cyan for 5); and the linestyle designates the sign of the superscript (solid for +, dashed for −).

As mentioned in Section 2, an important aspect of applying this symmetry-adapted PDF analysis is the choice of PDF peak width function. We compare results from three of these functions in Fig. 4 ▸ [beq_r_r2, beq_spherical and beq_PDFfit2, the *TOPAS* implementation of the *PDFgui* (Farrow *et al.*, 2007 ▸) peak shape function]. The three functions produce quite similar results – the ‘ranking’ of the irreps at each temperature is reasonably consistent. The function that performs the best for this compound is beq_r_r2 – the trend for each irrep is quite smooth and there are none of the erratic jumps in *R*_w_ that can be seen in both of the other functions. Since in this case the coefficient of *r* ^2^ was fixed at zero, this function also has only two parameters per site, which is the same as the *PDFfit* function used and the spherical function. We now go on to analyse the results of this method.

The distortions used in this analysis can be sorted into three general types: rigid unit modes, which consist of coherent rotations of the octahedra; semi-rigid ‘scissoring’ modes, where there is a scissoring of some of the Sc—F bond angles within the octahedra; and bond-stretching modes, where some *M*—F bond lengths change. Most irreps only have one of these types of distortion associated with them, although some have two. In Fig. 4 ▸, the irreps are clearly separated into two ‘bands’, one fitting well and one fitting poorly. The band of poorly fitting irreps all have distortions with a bond-stretching character, which are typically the highest-energy modes, meaning they have little influence on the local structure. The irreps in the other band all have at least one distortion associated with them that is of either rigid or semi-rigid unit mode character. There are four zone-boundary irreps that consistently have the lowest weighted *R* factors and have the greatest amplitudes: X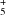, X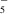, M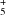 and M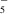. All four of these have one distortion associated with them that is of scissoring-mode character. All four modes have an amplitude (normalized to the supercell) between 0.9 and 1.0 Å and vary linearly with temperature to values between 1.30 and 1.45 Å at 450 K. There are a few possible conclusions one could make from this information, which would need further analysis to explore.

By performing competitive two-phase refinements between scissoring modes and RUMs, we found that scissoring modes dominate the motion of the fluorine ions (Bird *et al.*, 2020 ▸). This could mean that the negative thermal expansion in ScF_3_ arises purely from these kind of motions, and Wendt *et al.* (2019 ▸) have even argued that the fluorine ion motions are predominately uncorrelated. Alternatively, it could mean that these scissoring modes act in conjunction with the RUMs to produce the observed NTE. A low energy cost for scissoring-type deformations of the octahedra would increase the proportion of quasi-RUMs, modes of mixed RUM and octahedral deformation character, with negative Grüneisen parameters. Both of these possibilities are analysed further in our (Bird *et al.*, 2020 ▸) and others’ recent work (Dove, 2019 ▸; Dove *et al.*, 2020 ▸).

### 3.2. Barium titanate   

Barium titanate (BaTiO_3_) is one of the most well known ferroelectric materials. The Curie temperature (*T*_C_) for BaTiO_3_ is 393 K, above which the material has the archetypal cubic perovskite structure. Below *T*_C_, the structure is distorted into a *P*4*mm* tetragonal phase and, because of this, the cubic-to-tetragonal distortion was initially discussed in terms of displacive phase transitions (Cochran, 1959 ▸). Two lower-temperature phases, an orthorhombic *Amm*2 phase and a rhombohedral *R*3*m* phase, were discovered, with transition temperatures of 278 and 183 K, respectively (Kay & Vousden, 1949 ▸; Rhodes, 1949 ▸). The existence of these phases is inconsistent with the picture of second-order displacive phase transitions. This anomaly, in conjunction with the observation of diffuse scattering in all but the rhombohedral phase (Comes *et al.*, 1968 ▸), led to the development of an order–disorder model for BaTiO_3_ (Comès *et al.*, 1970 ▸). In this example, we aim to show that this method is sensitive to the nature of the local displacements in BaTiO_3_.

**Figure 5:**
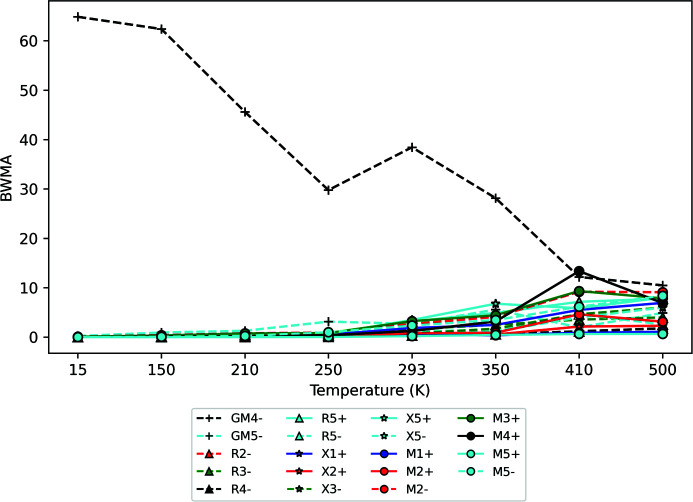
A plot showing the BWMAs for BaTiO_3_. The data points are made to differentiate between irrep labels: the marker designates the *k* point (a circle for M, a triangle for R, a star for X and a plus for Γ); the colour designates the number in the subscript (blue for 1, red for 2, green for 3, black for 4 and cyan for 5); and the linestyle designates the sign of the superscript (solid for +, dashed for −).

We generate the distortion modes using the high-symmetry 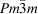 structure for BaTiO_3_, with the setting Ba 1*a* (0, 0, 0); Ti 1*b* (1/2, 1/2, 1/2); O 3*c* (0, 1/2, 1/2). The beq_r_r2 function is used to account for correlation of displacements. The PDFs used for this study were generated from total scattering data collected on the GEM instrument at the ISIS neutron and muon source. *Q*_max_ = 40 Å^−1^ was used with d*Q* = 0.033 Å^−1^. Further experimental details can be found in our previous publication on the subject (Senn *et al.*, 2016 ▸). An example input file is included in the online repository (Bird & Senn, 2021 ▸). Since the structure undergoes phase transitions, we give different constraints for the lattice parameters and angles depending on the average structure. For example, when the structure is cubic, we restrict all three lattice parameters to be equal, whereas they are allowed to be different in the orthorhombic structure. We use a different method to view the initial results than for ScF_3_, where we simply viewed the best fit for each irrep at each temperature. For BaTiO_3_ we are more interested in the primary order parameter. Therefore, we calculate the mode amplitude for each refinement and weight it according to a Boltzmann distribution exp[(*R*_w global_ − *R*_w_)/σ], where *R*_w_ is the weighted phase *R* factor for that refinement, *R*_w global_ is the lowest value for *R*_w_ across all irreps for each temperature and σ is the value of a meaningful difference in *R*_w_, taken to be 0.8%. We then sum this value for each irrep and obtain what we term a Boltzmann weighted mode amplitude (BWMA). These BWMAs are plotted in Fig. 5 ▸. The mode amplitudes themselves are calculated for each refinement by first dividing the amplitude of each mode that enters into the irrep by the normalization factor (given in the *ISODISTORT* CIF), putting it on an absolute, rather than fractional, scale. Subsequently, the square root of the sum of squares of the individual normalized mode amplitudes is taken as the overall mode amplitude for the irrep.

We can classify the behaviour of the BWMAs into three different groups: the first has low values in the cubic phase with larger values at lower temperatures; the second has small values in the cubic phase which drop down to near zero at lower temperatures; and the third has near-zero values for all temperatures. We can disregard all those in the third group, as they clearly do not have a significant contribution to the local symmetry-breaking distortions in BaTiO_3_. There is only one irrep in the first group, the 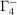 irrep. The modes belonging to this irrep are displacements of the Ti and O atoms (note that *ISODISTORT* additionally includes displacements of the Ba atoms in this irrep, but we fix these at zero to avoid a floating origin of the unit cell) and are clearly the order parameters relevant for the ferroelectric phase transitions. The second group of irreps have modes which are soft in the cubic phase, since they have similar BWMAs to the primary order parameter in that phase. The two irreps with the greatest BWMAs in the cubic phase are X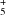 and M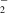, which are known to be soft eigenvectors of the system and are on the same line in the phonon dispersion curves as 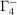. An interesting comparison between the results for BaTiO_3_ and ScF_3_ can be made here – both analyses pull out X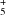 as a mode of interest, but the overall character of the distortion is different in each case. For ScF_3_, the distortion is mostly of the scissoring-mode character, with insignificant contributions from the other modes. In BaTiO_3_, the main distortion is the anti-ferroelectric displacements.

We now move on to analyse the underlying symmetry of the order parameter 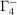. Each mode belonging to 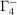 has three branches, and hence has a general order parameter direction (OPD) (*a*, *b*, *c*). This general distortion would reduce the symmetry of the structure to *P*1. A more constrained OPD would break fewer symmetry operations. The relevant order parameters for 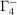 are (*a*, 0, 0), resulting in a *P*4*mm* space group, (*a*, *a*, 0) (*Amm*2), (*a*, *a*, *a*) (*R*3*m*), (*a*, *b*, 0) (*Pm*) and (*a*, *a*, *b*) (*Cm*). In the tetragonal and orthorhombic phases, the atoms tend to have an (*a*, *a*, *b*) OPD. This initially seems to reveal an underlying monoclinic symmetry in the displacements. However, this may also be viewed as a local rhombohedral distortion split by the global lattice distortion. This is consistent with the order–disorder model of the phase transitions in BaTiO_3_.

## 4. Summary   

In conclusion, we have demonstrated in detail how to perform the symmetry-adapted pair distribution function analysis technique with the *TOPAS-Academic* software v6. We have also provided two applications of this technique, with example input files so the reader can reproduce the above results as an introduction the technique. It has been demonstrated to be a useful technique to gain insight into both dynamic and static distortions in perovskite and perovskite-related materials. It is envisaged that use of this approach in conjunction with the freely available scripts (provided via GitHub) will enable other researchers to robustly and routinely evaluate lattice dynamics and local distortions of other solid-state materials.

## Supplementary Material

Scripts, input file and data required to reproduce the example discussed in the main manuscript.: https://doi.org/10.5281/zenodo.5036824
